# Characterization of the Circumlimbal Suture Model of Chronic IOP Elevation in Mice and Assessment of Changes in Gene Expression of Stretch Sensitive Channels

**DOI:** 10.3389/fnins.2017.00041

**Published:** 2017-02-10

**Authors:** Da Zhao, Christine T. O. Nguyen, Vickie H. Y. Wong, Jeremiah K. H. Lim, Zheng He, Andrew I. Jobling, Erica L. Fletcher, Holly R. Chinnery, Algis J. Vingrys, Bang V. Bui

**Affiliations:** ^1^Department of Optometry and Vision Sciences, University of MelbourneParkville, VIC, Australia; ^2^Department of Anatomy and Neuroscience, University of MelbourneParkville, VIC, Australia

**Keywords:** glaucoma, intraocular pressure, eNTDPase, TRPV, P2X7, pannexin, electroretinography, retinal ganglion cells

## Abstract

To consider whether a circumlimbal suture can be used to chronically elevate intraocular pressure (IOP) in mice and to assess its effect on retinal structure, function and gene expression of stretch sensitive channels. Anesthetized adult C57BL6/J mice had a circumlimbal suture (10/0) applied around the equator of one eye. In treated eyes (*n* = 23) the suture was left in place for 12 weeks whilst in sham control eyes the suture was removed at day two (*n* = 17). Contralateral eyes served as untreated controls. IOP was measured after surgery and once a week thereafter. After 12 weeks, electroretinography (ERG) was performed to assess photoreceptor, bipolar cell and retinal ganglion cell (RGC) function. Retinal structure was evaluated using optical coherence tomography. Retinae were processed for counts of ganglion cell density or for quantitative RT-PCR to quantify purinergic (*P2x7, Adora3, Entpd1*) or stretch sensitive channel (*Panx1, Trpv4*) gene expression. Immediately after suture application, IOP spiked to 33 ± 3 mmHg. After 1 day, IOP had recovered to 27 ± 3 mmHg. Between weeks 2 and 12, IOP remained elevated above baseline (control 14 ± 1 mmHg, ocular hypertensive 19 ± 1 mmHg). Suture removal at day 2 (Sham) restored IOP to baseline levels, where it remained through to week 12. ERG analysis showed that 12 weeks of IOP elevation reduced photoreceptor (−15 ± 4%), bipolar cell (−15 ± 4%) and ganglion cell responses (−19 ± 6%) compared to sham controls and respective contralateral eyes (untreated). The retinal nerve fiber layer was thinned in the presence of normal total retinal thickness. Ganglion cell density was reduced across all quadrants (superior −12 ± 5%; temporal, −7% ± 2%; inferior −9 ± 4%; nasal −8 ± 5%). Quantitative RT-PCR revealed a significant increase in *Entpd1* gene expression (+11 ± 4%), whilst other genes were not significantly altered (*P2x7, Adora3, Trpv4, Panx1*). Our results show that circumlimbal ligation produces mild chronic ocular hypertension and retinal dysfunction in mice. Consistent with a sustained change to purinergic signaling we found an up-regulation of Entpd1.

## Introduction

Glaucoma is the leading cause of irreversible blindness in the world (Quigley and Broman, 2006) and trails only cataract and uncorrected refractive error when reversible blindness is considered. It is a progressive disease of the optic nerve that generally leads to selective loss of retinal ganglion cells (RGCs), the output cells of the eye. The most widely studied and perhaps best-understood risk factor for glaucoma is elevated intraocular pressure (IOP). However, despite a significant body of work, our understanding of the mechanisms by which chronic IOP elevation leads to ganglion cell injury remains incomplete (Almasieh et al., 2012).

Significant insight into the pathogenic mechanisms of IOP-induced injury has arisen from both genetic (Libby et al., 2005; May and Mittag, 2006; Zhang et al., 2007; Lu et al., 2015) and inducible models of chronic pressure elevation in common laboratory animals (Pang and Clark, 2007; Morrison et al., 2008; Bouhenni et al., 2012). Inducible murine models of IOP injury are particularly useful as they allow the mechanistic pathways involved in IOP-induced injury to be explored via the use of genetic manipulation (Pang and Clark, 2007; Morrison et al., 2008; Cone et al., 2010). For example, chronic IOP elevation has been generated in transgenic mice lacking the chemokine receptor Cx3cr1, affording insight into the role that Cx3cr1-mediated microglial activation has in this neurodegenerative process (Wang et al., 2014). Furthermore, the use of transgenic mice expressing green fluorescent protein within RGCs has allowed the examination of early dendritic changes in response to IOP elevation (El-Danaf and Huberman, 2015).

In mice, a range of approaches have been used to chronically increase IOP over weeks to months. Studies have targeted episcleral venous outflow via cauterization (Ruiz-Ederra and Verkman, 2006), and laser ablation (Mabuchi et al., 2004; Holcombe et al., 2008), while intracameral injections (into the anterior chamber) of inert materials to impede trabecular outflow have also been undertaken (Benozzi et al., 2002; Moreno et al., 2005; Morrison et al., 2008; Sappington et al., 2009; Frankfort et al., 2013; Bunker et al., 2015). Such approaches often require more than a single manipulation to create a chronic mild IOP elevation that mimics the human condition of ocular hypertension (Cone et al., 2012). Whilst a range of exogenous materials has been injected into the anterior chamber (Weber et al., 1998; Charng et al., 2013; Kezic et al., 2013), such manipulations are not without potential confounds. Kezic et al. (2013) showed that simply cannulating the mouse anterior chamber without introducing any foreign material initiates inflammatory processes. Another potential issue with the introduction of foreign material is that it can impede the optics of the eye making *in vivo* electrophysiology and imaging difficult. This may be a particular challenge for longitudinal *in vivo* imaging of cells in reporter mouse strains, such as the ganglion cell Thy1-yellow fluorescent protein mouse (Lindsey et al., 2015).

Recently our group Liu et al. (2015) extended the approach of Joos et al. (2010) to induce IOP elevation by attaching a simple circumlimbal suture at 5–6 subconjunctival anchor points to fix the suture in place around the rat eye just behind the limbus. This approach produced stable mild long-term IOP elevation of about 7–10 mmHg (or 50–65% higher than normal) consistent with human ocular hypertension. After 12 weeks, we find a pattern of preferential RGC dysfunction (non-invasive electroretinography) and loss, with associated retinal nerve fiber layer thinning. As these data reflect retinal changes during glaucoma, this suture-based paradigm provides a useful model in which to evaluate glaucoma development. Moreover, this model has the advantage that the suture can be removed at any stage, thereby returning IOP to baseline without confounds related to IOP-lowering drug intervention. These advantages enable the study of neuroplasticity during phases of chronic injury (IOP elevation) and recovery (IOP normalization) (Liu et al., 2015). Although recovery from acute IOP elevation can be studied in various murine strains (Kong et al., 2011, 2012) it is currently difficult to study recovery from prolonged periods of IOP elevation. Hence, there are advantages in optimizing the circumlimbal suture model, successfully used in rats, for the mouse eye.

Significant advances have been made in understanding how stress resultant from chronic IOP elevation can lead to ganglion cell injury. There is compelling evidence that activation of stretch sensitive channels leads to the release of adenosine triphosphate (ATP) via pannexin channels, that aids the ganglion cell response to stress (Krizaj et al., 2014). Furthermore, purinergic signaling has been shown to transduce mechanical strain in a range of tissues (Burnstock, 2016) and play a critical role in IOP-induced changes in RGCs (Grygorczyk et al., 2013). In particular, extracellular ATP (Zhang et al., 2007; Reigada et al., 2008; Lu et al., 2015) is thought to be released from both RGCs and astrocytes via pannexin channels (Dvoriantchikova et al., 2012; Xia et al., 2012; Beckel et al., 2014) or via mechanosensitive-transient receptor potential (TRP) channels such as vanilloid (TRPV1, TRPV2, TRPV4) and ankyrin (TRPA1) channels (Egbuniwe et al., 2014; Sato et al., 2015). Although this activation of mechanosensitive channels/purinergic pathway may aid ganglion cells with their response to stress, excessive release of ATP and activation of P2X7 receptors can lead to cell death (Zhang et al., 2005; Resta et al., 2007). In order to limit extracellular ATP, several ecto-nucleoside triphosphate diphosphohydrolases (eNTDPase) phosphorylate ATP to adenosine (Iandiev et al., 2007) which has a protective effect in pressure-induced injury via its action on P1 receptors (A1, A2, A3) (Larsen and Osborne, 1996; Zhang et al., 2006). Recently, Lu et al. (2015) found an increase in *Entpd1* expression in three models of chronic IOP elevation, consistent with an ATP-dependent feedback mechanism.

Thus, the aims of this study are to consider if (1) the circumlimbal suture approach can also be used in mouse eyes to produce chronic ocular hypertension and (2) if this leads to changes in retinal function (ERG), retinal structure (optical coherence tomography) and ganglion cell density that recapitulates key features of glaucoma. (3) We also consider the effect that chronic IOP elevation has on gene expression of a number of mediators of mechanosensitivity and purinergic signaling, including *Trpv4, pannexin-1, P2x7, A3*, and *Entpd1*.

## Materials and methods

### Animals

All experimental procedures comply with the National Health and Medical Research Council Australian Code of Practice for the care and use of animals for scientific purposes and the Association for Research in Vision and Ophthalmology's Statement for the Use of Animals in Ophthalmic and Vision Research. Animal Ethics approval was issued from the Howard Florey Institute Animal Experimentation Ethics Committee (13-068-UM).

Adult male C57BL/6J mice were used and experimentation commenced at 12 weeks of age. All mice were obtained from the Animal Resources Centre (Canning Vale, WA, Australia) and housed with unrestricted access to normal chow (Barastoc, Melbourne, VIC, Australia) and water at the Melbourne Brain Centre (Kenneth Myer Building, Parkville, VIC, Australia). Room temperature was maintained at 21°C and lighting on a 12-h light/12-h dark cycle (maximum 50 lux inside the cage).

Animals were anesthetized with isoflurane (IsoFlo, Abbott, North Chicago, IL) for suture application. All other ocular assessments were conducted after intra-peritoneal injection of ketamine:xylazine (80:10 mg/kg, intra-peritoneal, Troy Laboratory, Glendenning, NSW, Australia). Corneal anesthesia and mydriasis were achieved using topical Alcaine (0.5% proxymetacaine, Alcon Laboratories, Sydney, NSW, Australia) and Mydriacyl (0.5% tropicamide, Alcon Laboratories), respectively. Body temperature was maintained at 37.5 ± 0.5°C using a heating pad.

### IOP manipulation and measurement

Forty mice were included in the 12 week trial, comprising an ocular hypertension (OHT) group (*n* = 23) and a sham group (suture removed after 2 days, *n* = 17). In all animals, one eye was randomly chosen to be the treated eye, and the contralateral eye served as a within animal untreated control.

**Figure 1:**
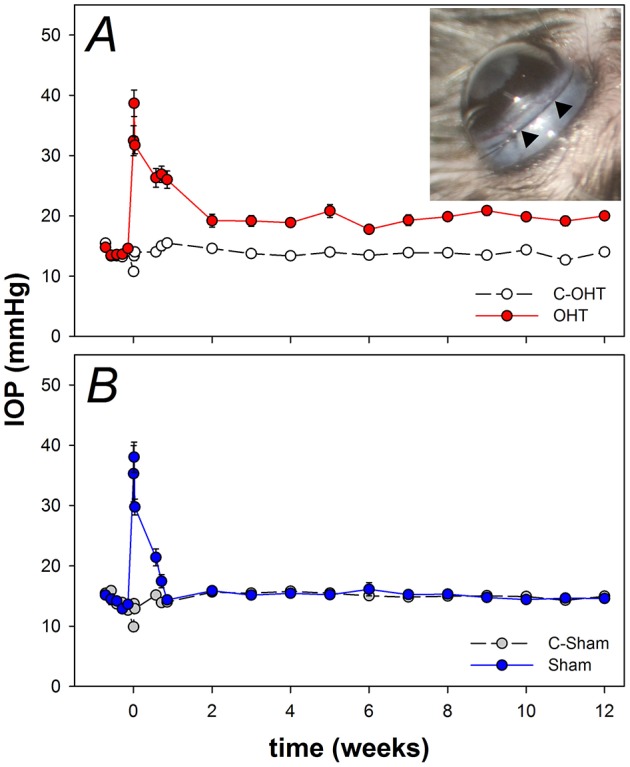
**Chronic intraocular pressure (IOP) elevation in mouse eyes. (A)** Group IOP (mean ± standard error of the mean) in ocular hypertensive (OHT, red *n* = 23) and their untreated contralateral control eyes (C-OHT, unfilled). Inset: Image of a mouse eye with a circumlimbal suture (arrowheads) in place. **(B)** IOP in eyes that had the suture removed on day 2 (Sham, blue *n* = 17) and their untreated contralateral control eyes (C-Sham, gray).

The circumlimbal suture method was adapted from our previous work in rats (Liu et al., 2015, 2016). Briefly, under anesthesia (isoflurane: 4% induction and 1.5% maintenance at 1.5 L/min) a 10/0 nylon suture is threaded underneath the conjunctiva at 4–5 evenly spaced locations around the globe (anchor points) preventing suture movement. In order to improve long-term stability at each anchor point, the suture was threaded underneath the conjunctiva as far as possible, taking care to ensure that the eye was not perforated. In doing so, the suture was buried under the conjunctiva 1 mm behind the limbus, evenly applying pressure onto the eyeball (Figure 1 insert). To adjust tension around the eye a slipknot was used. Throughout the procedure the cornea was hydrated with normal saline. In the OHT group, the suture remained in place for the duration of the experiment (12 weeks).

The sham surgical procedure was similar to the OHT group; with the exception that the suture was removed at day 2 post-surgery. This time point was chosen for suture removal as a circumlimbal suture in rats (Liu et al., 2015) produced an initial moderate IOP spike lasting for up to 2 days. By removing the suture at day 2 (Sham group) we could evaluate the effect of the initial IOP spike. The difference between the OHT and sham groups should then reflect the effect of the period of chronic IOP elevation only, i.e., ocular hypertension.

IOP was monitored in each eye over the 12 weeks using a rebound tonometer (Tonolab, iCare, Helsinki, Finland), and the average of 10 readings was returned as the IOP at each time point. Baseline IOP was the average of daily readings made over the 5 days before surgery. At the time of surgery, IOP was measured immediately after tightening the suture (2 min), as well as at 1 and 3 h after surgery. IOP was monitored daily for the first 3 post-operative days and then weekly until week 12. Except for the 2-min time point immediately following surgery, IOP was measured in awake animals without topical anesthesia. To minimize diurnal fluctuation, IOP measurement was conducted at the same time of day and under the same light conditions (150 lux at the bench top).

In both OHT and sham groups, structural and functional assessments were performed 12 weeks after surgery. Retinal function and structure (OHT *n* = 23 and sham *n* = 17) were measured in both eyes of all animals, and subgroups were randomly allocated for histology (OHT *n* = 12, sham *n* = 8 animal) and PCR (OHT *n* = 9, sham *n* = 9 animals).

### Electroretinography

Dark-adapted full-field ERG was utilized to examine retinal function as described previously (Bui and Fortune, 2004; Lim et al., 2014; Liu et al., 2015). Mice were dark-adapted overnight (12 h) prior to recording (Behn et al., 2003), and light exposure was minimized during set-up to ensure maximal retinal sensitivity and optimize ganglion cell-specific scotopic threshold response (STR) measurement (Bui and Fortune, 2004). A pair of custom-made chlorided silver (99.9%, A&E Metal Merchants, Sydney, NSW, AU) active and ring-shaped reference electrodes, connected to platinum leads (F-E-30, Grass Telefactor, West Warwick, RI), were placed on the central cornea and sclera, respectively. A stainless steel needle electrode (F-E2-30, Grass Telefactor) was inserted subcutaneously into the tail to act as the ground. Responses were measured from both eyes at the same time.

Calibrated (IL1700, International Light Technologies, Peabody, MA) light stimuli were delivered across a range of energies from −6.35 to 2.07 log cd·s/m^2^ by an array of 8 white light emitting diodes (LEDs, 8 Watt Luxeon LED, Philips Lumileds Lighting Company, San Jose, CA) and a dim LED (0.1 Watt Luxeon LED, Philips Lumileds Lighting Company) embedded inside a Ganzfeld sphere (Photometric Solutions International, Oakleigh, VIC, Australia). Signals were acquired with Scope™ software (ADInstruments Pty Ltd., Bella Vista, NSW, Australia) at a 4-kHz sampling rate with pre-amplifier (P511, Grass Telefactor, Natus, Pleasantown, CA) and hardware band-pass filter settings of 0.3–1000 Hz (−3 dB). Signals were digitized (ML785 Powerlab 8SP, ADInstruments) and saved for *post-hoc* processing.

The very first electronegative component of the ERG waveform (the a-wave) in response to the brightest stimuli was modeled using a delayed-Gaussian function to expose the P3 component (Lamb and Pugh, 1992; Hood and Birch, 1994). The maximal amplitude (designated RmP3) returned by this model reflects photoreceptor function (number, outer segment length, or density of non-specific cationic channels). The second and largest component of the ERG waveform (the b-wave) is the combination of the photoreceptoral P3 and a P2 component, which is known to reflect ON-bipolar cell activity. We subtract the P3 model from the raw ERG waveform to reveal the putative P2. The amplitude of each P2 waveform was plotted as a function of intensity and modeled with classical agonist-receptor characteristics using a hyperbolic curve (Fulton and Rushton, 1978). This model returns a maximum amplitude, which provides an index of bipolar cell integrity. The small high frequency wavelets that overlie the P2 leading edge, known as oscillatory potentials (OPs) and can be isolated from the P2 using a digital band-pass filter (50–180 Hz, −3 dB), to provide an index of amacrine cell derived inner retinal inhibitory pathways. Finally, the positive STR (pSTR), which can be measured near absolute light threshold is a good indicator of RGC function in rodents (Saszik et al., 2002; Bui and Fortune, 2004). To improve the signal to noise ratio, peak pSTR amplitude and its implicit time were averaged from parameters isolated from waveforms collected at −4.9, −5.01, and −5.31 log cd·s/m^2^.

### Optical coherence tomography

Immediately after ERG recordings spectral domain optical coherence tomography (SD-OCT, Envisu R2200 VHR, Bioptigen Inc., Durham, NC) was utilized to image structural changes in living eyes. Both the anterior segment and cross sections of the posterior retina through the center of the optic nerve were evaluated as described previously (Chinnery et al., 2015).

Anesthetized mice were placed on a rodent alignment stage and a drop of ocular lubricant (Systane, Alcon Laboratories) was applied to improve tear film optics for imaging. Once the central apex was aligned with the objective lens, volumetric 4 x 4 mm rectangular scans (1000 A-scans per B-scans, 100 horizontal B-scans evenly spaced in the vertical dimension) of the anterior segment were captured using an 18-mm telecentric lens (Envisu R2200, Bioptigen Inc.). From en face images pupil size (PS) was determined by the average of horizontal (180°) and vertical (90°) width. Anterior chamber depth (ACD) and trabecular meshwork-iris angle (TIA) were quantified in B-scans that were centered at the corneal apex.

Retinal OCT images were captured using a mouse objective (Envisu R2200, Bioptigen Inc.). A 2.5 × 2.5 mm volume scan was acquired with a depth of 1.7 mm (1000 A-scans per B-scans, 200 horizontal B-scans evenly spaced in the vertical dimension). Four B-scans that crossed the optic nerve head (ONH) were analyzed using FIJI software (https://fiji.sc/) available in the public domain. In each B-scan the inner limiting membrane, retinal nerve fiber layer (RNFL), inner plexiform layer and Bruch's membrane were manually segmented by a masked observer. From these segments, RNFL thickness and total retinal thickness (TRT) excluding the ONH region were averaged over a distance of 100–800 μm on either side of the ONH to return an average thickness for each layer. In addition, Bruch's membrane opening (BMO) width was measured along with the minimum rim thickness (MRT), which is the shortest distance from Bruch's membrane opening to the inner limiting membrane (surface of the retina) and comprises retinal nerve fibers as they turn to exit the eye. The final parameters for each eye represent the average of measurements made for both temporal and nasal retina across all 4 B-scans.

After acquiring retinal OCT images, retinal blood flow was determined using an annular Doppler blood flow B-scan (1000 A-scans, Bioptien Inc.), located 1 mm from the center of the ONH. Regions showing a significant Doppler shift are indicated by red and blue pixels. Red pixels represent arterial flow (toward the objective), whereas blue pixels indicate venous flow (away from the objective). Thus, counting the number of pixels provides a surrogate measure of blood flow and arterial or venular size. Both red and blue signals were extracted using a thresholding algorithm available with FIJI software (https://fiji.sc/) and summed to return blood flow in arbitrary units (au).

In addition, fundus photos were acquired using the Micron III small animal imaging platform (Phoenix Research Labs, Pleasanton, CA, USA) to ensure that the fundus was unaffected by 12 weeks of experimentation.

### Histological assays

Following ERG and OCT measurement, in subset of animals from each group (OHT *n* = 12, and sham *n* = 8), eyes were removed and a mark was created on the superior limbus using a surgical cautery iron to retain orientation of the eye. Whole eyes were fixed in 4% paraformaldehyde in 0.1 M phosphate buffer (PB) for 1 h at room temperature. Retinae were dissected in flat mount, the superior quadrant marked with a radial cut, washed with PB, incubated with 20 mM EDTA for 1 h at 37°C then blocked in PB containing 3% BSA, 0.3% triton X-100 and 5% goat serum. Retinae were incubated overnight at 4°C with an antibody to RNA-binding protein with multiple splicing (RBPMS) to identify ganglion cells (1:1000, Invitrogen, Thermo Fisher Scientific, Carlsbad, USA). RBPMS has been shown to be a robust marker of RGCs in mice (Rodriguez et al., 2014). Retinae were washed in PB and subsequently incubated with goat anti-rabbit Alexa Fluor-647 (1:500; Invitrogen) for 1 h at room temperature. Flat mounts were counterstained (Hoechst; 1:1000, Roche Applied Science, Mannheim, Germany), washed and cover slipped photoreceptor side down.

Images were acquired using a confocal (Leica SP8, Leica Microsystems, Wetzlar, Germany) microscope (40x) from central (0.3 mm from optic nerve) and peripheral (0.3 mm from the retinal-ciliary body border) eccentricities in all four retinal quadrants (superior, temporal, nasal, and inferior). A total of eight confocal z-stacks were collected for each eye (Leica SP8, Leica Microsystems). Z-series through the ganglion cell layer were collected at 1 μm steps using a 512 × 512 scan format (290.62 × 290.62 μm). Ganglion cell densities were manually counted by a masked observer using FIJI software.

### Quantitative real-time polymerase chain reaction

A subgroup of animals (OHT *n* = 9 and sham groups *n* = 9) were terminally anesthetized, and the eye was immediately enucleated, the retina isolated from the posterior eyecup, and stored at −80°C until use. Quantitative real-time polymerase chain reaction (qRT-PCR) was performed as described previously (Jobling et al., 2013; Ho et al., 2015). Briefly, total RNA was isolated from retina using commercial spin columns (RNeasy, Qiagen, Valencia, CA) incorporating DNase I treatment to remove genomic contamination. Retinal samples and dilutions of external gene-specific standards were reverse transcribed (250 ng, Tetro, Bioline, London, UK) and subsequently diluted to 5 ng/μL for quantitative PCR.

Using specific primers (Table 1) gene expression of several potentially pressure sensitive receptors was quantified on a Rotorgene V3000 (Corbett Research, Mortlake, NSW, Australia) using a SYBR-green based reaction mixture (SensiFast, Bioline, Alexandria, NSW, Australia). Negative controls and four-point standard curves were performed on every run. All standards and samples were amplified in triplicate and absolute gene copy number was calculated with reference to standard curves (Rotorgene V6.1 software; Corbett Research). Gene copy number was expressed normalized to the housekeeping gene hypoxanthine-guanine phosphoribosyltransferase (*Hprt*).

**Table 1:** **Gene specific oligonucleotide primer sequences**.

**Gene**	**Sequence**	**Forward primer**	**Reverse primer**	**Product size (bp)**
*P2x7*	NM_011027.2	gcttgggaaaagtctgcaag	gacttaggggccacctcttc	162
*Adora3*	NM_009631.4	gttccgtggtcagtttggat	gcgcaaacaagaagagaacc	216
*Panx1*	NM_019482.2	ggccacggagtatgtgttct	tacagcagcccagcagtatg	247
*Trpv4*	NM_022017.3	agaaaggtcgtggagaagca	atcagtcaggcgcttcttgt	174
*Entpd1*	NM_009848.3	catccaagcatcaccagact	atgatcttggcaccctggaa	154
*Hprt*	NM_013556.2	cctaagatgagcgcaagttgaa	ccacaggactagaacacctgctaa	86

*Mouse-specific primers were designed to published gene sequences. Oligonucleotides are shown 5′–3′ and product sizes are highlighted. For the generation of external standards primers contained a T7 promoter sequence at the 5′ end of the forward primer, while a poly T_15_ sequence was included at the 5′ end of the reverse primer*.

### Statistical analysis

Statistical analysis was conducted using Prism 6 software (GraphPad Software Inc., USA). Group data are given as mean ± SEM. Results for treated eyes (OHT and sham) are expressed relative to their own untreated contralateral control eyes. Normality (Shapiro-Wilk test) and homogeneity of variance (Bartlett's test) were established before employing paired *t*-tests and ANOVA. Comparisons between treated and control eyes were undertaken using paired *t*-tests. Comparison of control groups (C-OHT vs. C-Sham) was undertaken using unpaired *t*-test. Two-way repeated measures ANOVA was used to compare IOP between groups (OHT, sham, C-OHT, C-Sham) across the 12-weeks of experimentation.

## Results

### Chronic IOP elevation in the circumlimbal suture model

There was a moderate attrition rate associated with suture surgery. A 50% success rate (*n* = 40) resulted at day 2 from a total of 81 mice that underwent suture surgery. The most common cause of complication was hyphema (*n* = 29, 71%), followed by suture breakage, slippage or conjunctival tear (*n* = 12, 29%).

As previously reported for rats (Liu et al., 2015, 2016), the circumlimbal suture approach when applied to mouse eyes produced a similar pattern of IOP elevation. Over 5 days of baseline measurement, IOP in the ocular hypertension (OHT) group was stable at ~15 mmHg (Figure 1A). Suture application resulted in immediate IOP elevation, to 32.5 ± 2.5 mmHg at 2 min and 38.7 ± 2.2 mmHg at 1 h. On day 3 it returned to 26.0 ± 1.4 mmHg and by week 2 it had settled to 19.2 ± 1.1 mmHg (Figure 1A). From week 2 onwards IOP in the OHT eye was stable and remained ~5 mmHg higher than the untreated contralateral eye (19.5 ± 0.8 mmHg vs. 13.8 ± 0.5 mmHg, 41% increase, two-way RM ANOVA from weeks 2 to 12, interaction: *p* = 0.21, treatment: *p* < 0.01). Over these 12 weeks, IOP in the untreated contralateral eye did not change significantly (one-way ANOVA from weeks 2 to 12, *p* = 0.10).

Figure 1B shows that the sham group also had a significant IOP spike (35.3 ± 4.7 mmHg at 2 min, 38.0 ± 2.5 mmHg at 1 h), similar to that observed in the OHT group. Suture removal on day 2 returned IOP in sham eyes to normal levels (day 2, 17.5 ± 1.0 mmHg; day 3, 14.3 ± 0.6 mmHg) that were not significantly different from the untreated contralateral control eye for the remainder of the treatment period (15.2 ± 0.6 mmHg vs. 15.1 ± 0.5 mmHg, two-way ANOVA, interaction: *p* = 0.92, treatment: *p* = 0.97, time: *p* = 0.18). Additionally, IOP in sham control eyes was similar to that of the untreated control eyes of the OHT group (13.8 ± 0.2 mmHg vs. 14.4 ± 0.3 mmHg, *p* = 0.08).

### The effects of chronic circumlimbal suture on the anterior segment

**Figure 2:**
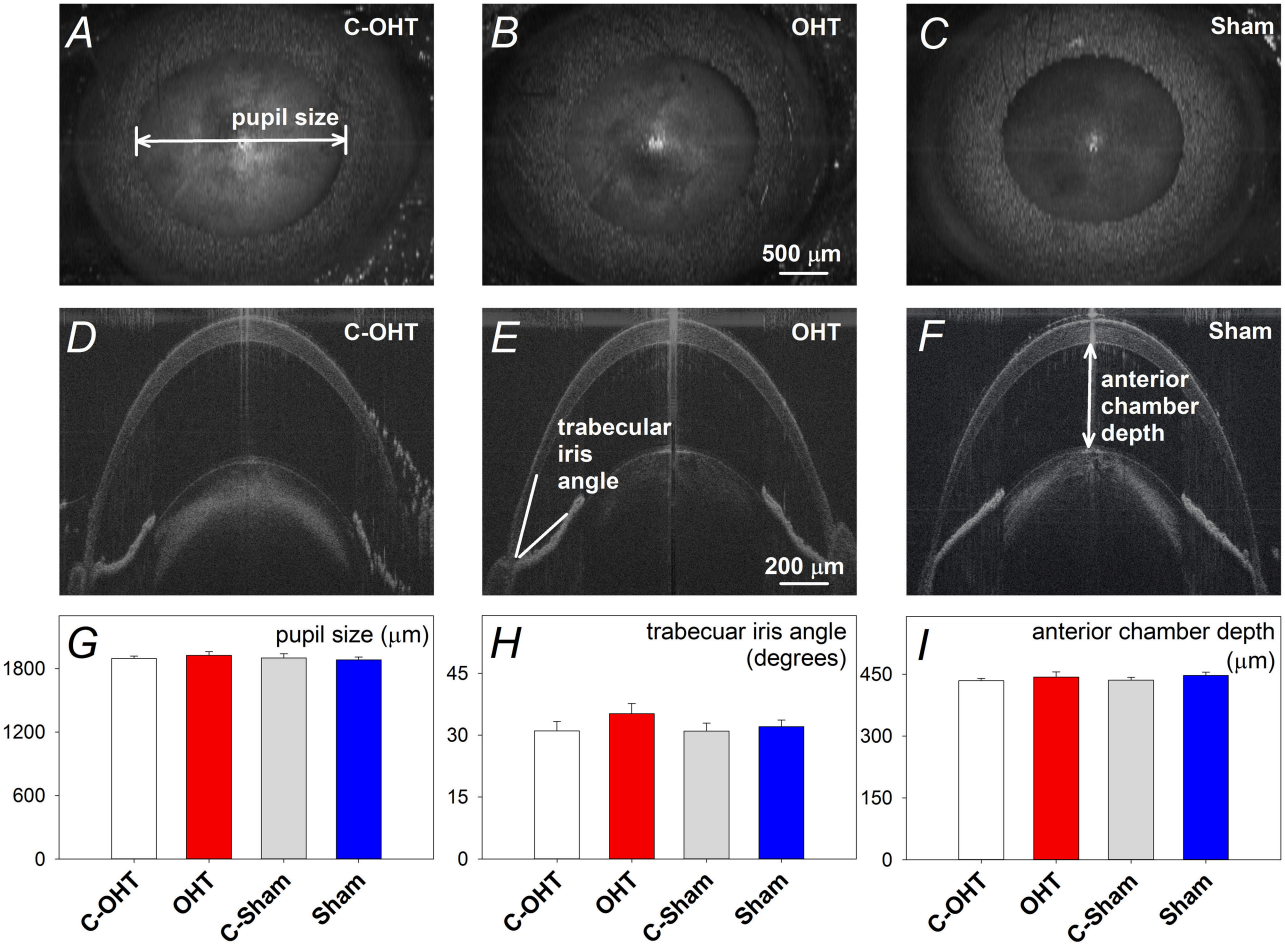
**Effect of circumlimbal suture and chronic intraocular pressure (IOP) elevation on the mouse anterior segment. (A–C)** Representative en face images of an ocular hypertensive (**B**. OHT), its fellow control (**A**. C-OHT) eye, and a sham operated eye (**C**. Sham) at week 12, acquired during anterior segment imaging allowing pupil diameter to be quantified. **(D–F)** Sagittal optical coherence tomography images of the anterior chamber for an OHT **(E)**, its control eye **(D)**, and a sham operated eye **(F)**. **(G)** Averaged (± SEM) pupil size for OHT (*n* = 23, red), C-OHT (unfilled) as well as Sham (*n* = 17, blue), and their contralateral control eyes (C-Sham, gray). **(H)** Average trabecular meshwork-iris angle (TIA). **(I)** Average anterior chamber depth.

Given the location of the suture it was important to rule out damage to the anterior segment given such damage is not found in primary open angle glaucoma. Figure 2 show representative images of pupil diameter acquired during anterior segment imaging for a control eye (C-OHT, Figures 2A,D), the contralateral treated eye (OHT, Figures 2B,E) and a sham operated eye (Sham, Figures 2C,F). After 12 weeks of chronic IOP there was no significant difference in pupil diameter between eyes (OHT 1926 ± 35 μm vs. C-OHT 1896 ± 22 μm, *p* = 0.39; Figure 2G). This was also the case for the sham group (sham and contralateral control eye, C-sham, *p* = 0.68). Additionally the anterior segment showed no significant change following suture application. Anterior chamber angle was not different (Figure 2H, *p* = 0.15) between OHT (Figure 2D, 35° ± 2°) and control eyes (Figure 2E, 31° ± 2°). Likewise, anterior chamber depth (ACD) was similar between the two eyes in the OHT group (Figure 2I, OHT 442.9 ± 12.7 μm vs. C-OHT 434 ± 5.5 μm, *p* = 0.46). There was no change in the anterior chamber angle (TIA) or anterior chamber depth (ACD) of the sham group (Figure 2H, TIA sham 32° ± 2° vs. TIA C-Sham 31° ± 2°, *p* = 0.70; Figure 2I, ACD sham 447 ± 8.3 μm vs. ACD C-Sham 435 ± 6.7 μm, *p* = 0.33).

### The effect of chronic circumlimbal suture on retinal blood flow

**Figure 3:**
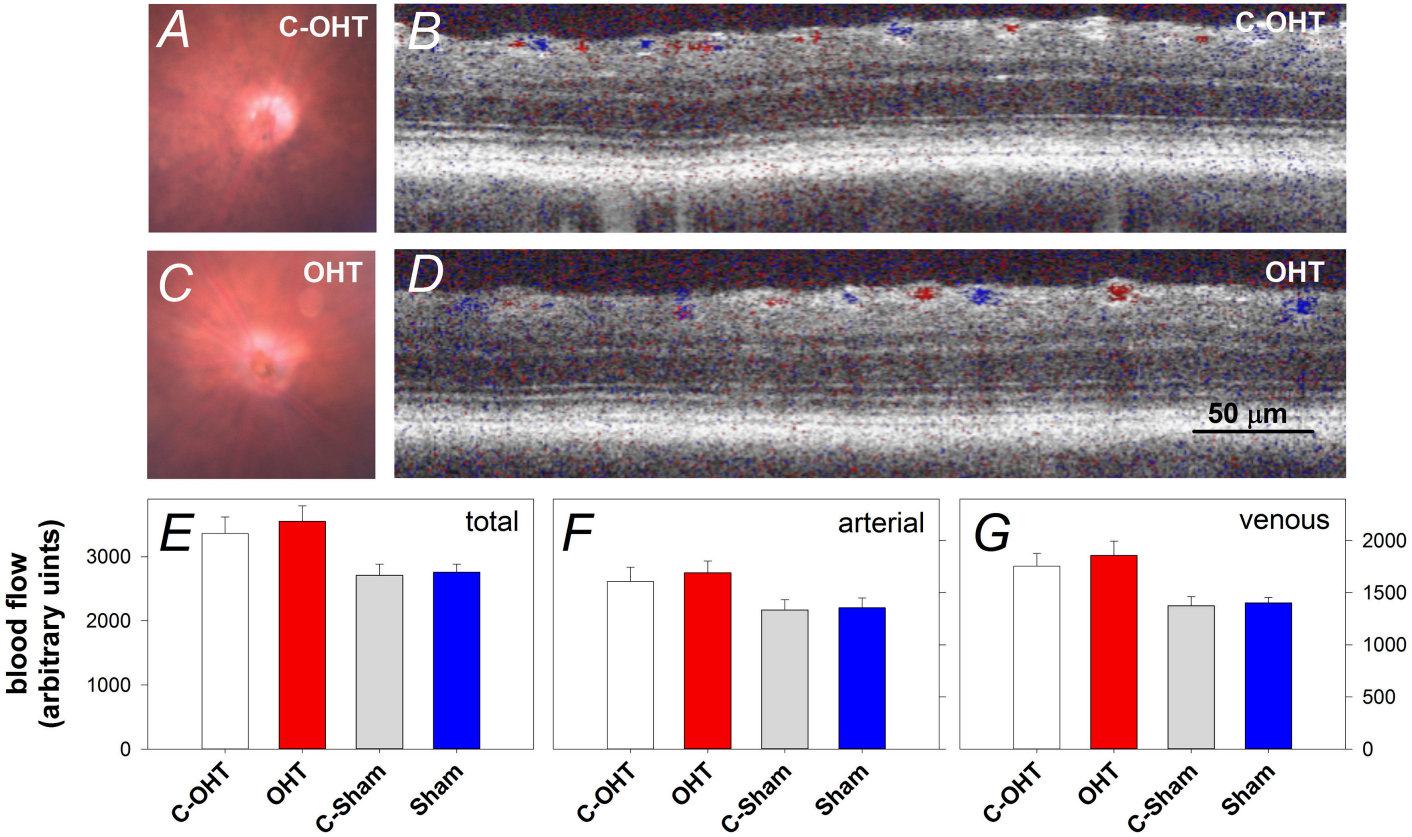
**Effect of chronic circumlimbal suture on retinal blood flow**. **(A,C)** Fundus images of a control and the treated eye in the OHT group. **(B,D)** Representative images of Doppler blood flow annular scan in the same C-OHT and OHT eyes. **(E)** Group average (± SEM) total blood flow signal in OHT (*n* = 23, red) and Sham (*n* = 17, blue) treated eyes and their contralateral controls (C-OHT unfilled, C-Sham gray). **(F)** Group average arterial signal. **(G)** Group average venous signal.

To ensure that the suture did not induce retinal ischemia, we assessed perfusion of the retinal vasculature via fundus photos and Doppler OCT. Representative fundus images in Figure 3 show that the retina was perfused in an OHT eye as well as its contralateral control eye (Figures 3A,C). For the same eyes, retinal blood flow was also examined using a Doppler annular scan. Consistent with fundus photography, robust arterial (red signal) and venous (blue signal) flow could be detected in eyes after 12-weeks of chronic IOP (Figures 3B,D). Blood flow quantification revealed no difference in total (Figure 3E, OHT 3548 ± 241 vs. C-OHT 3360 ± 258, *p* = 0.24), arterial (*p* = 0.28) or venous blood flow (*p* = 0.30) between OHT and contralateral control eyes. Similarly, sham eyes were not different from their controls (total 2757 ± 128 vs. 2707 ± 177, *p* = 0.8; arterial, *p* = 0.86; venous, *p* = 0.78). Total retinal blood flow as well as arterial and venous blood flow was higher in the OHT group compared with the sham group (one-way ANOVA, *p* < 0.01).

### The effect of chronic IOP elevation on retinal function

**Figure 4:**
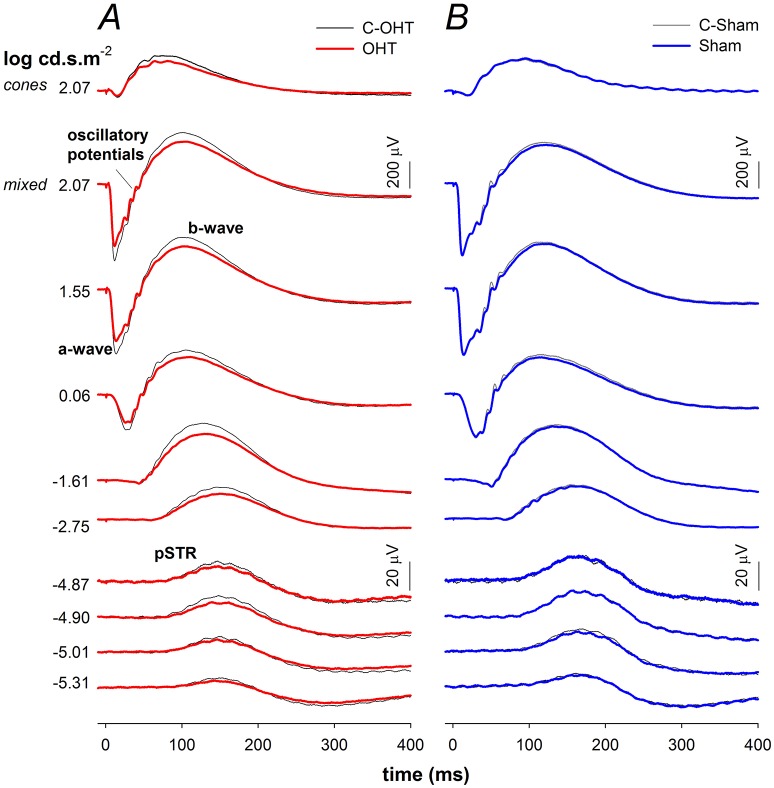
**Effect of chronic IOP elevation on ERG waveforms. (A)** Ocular hypertensive (OHT, red traces, *n* = 23) and control eyes (C-OHT, black traces) **(B)** Sham (blue traces, *n* = 17) and control eyes (C-Sham, black traces).

Next we considered the effect of chronic IOP elevation on the function of the major retinal neuronal classes using the ERG by comparing OHT and sham waveforms in Figures 4A,B respectively. The waveforms elicited using the dimmest light levels reflect ganglion cell dominated responses, known as the positive scotopic threshold response (pSTR). At moderate flash energies between −4.87 up to −1.61 log cd·s/m^2^ rod ON-bipolar cell responses dominate. At the brightest flashes, the response comprises both rod and cone contributions, with outer retinal function (photoreceptoral a-wave and ON-bipolar cell b-wave) being readily discernable. Although waveforms from sham treated and C-Sham eyes showed no obvious differences, there were differences evident in waveform obtained from the OHT group.

**Figure 5:**
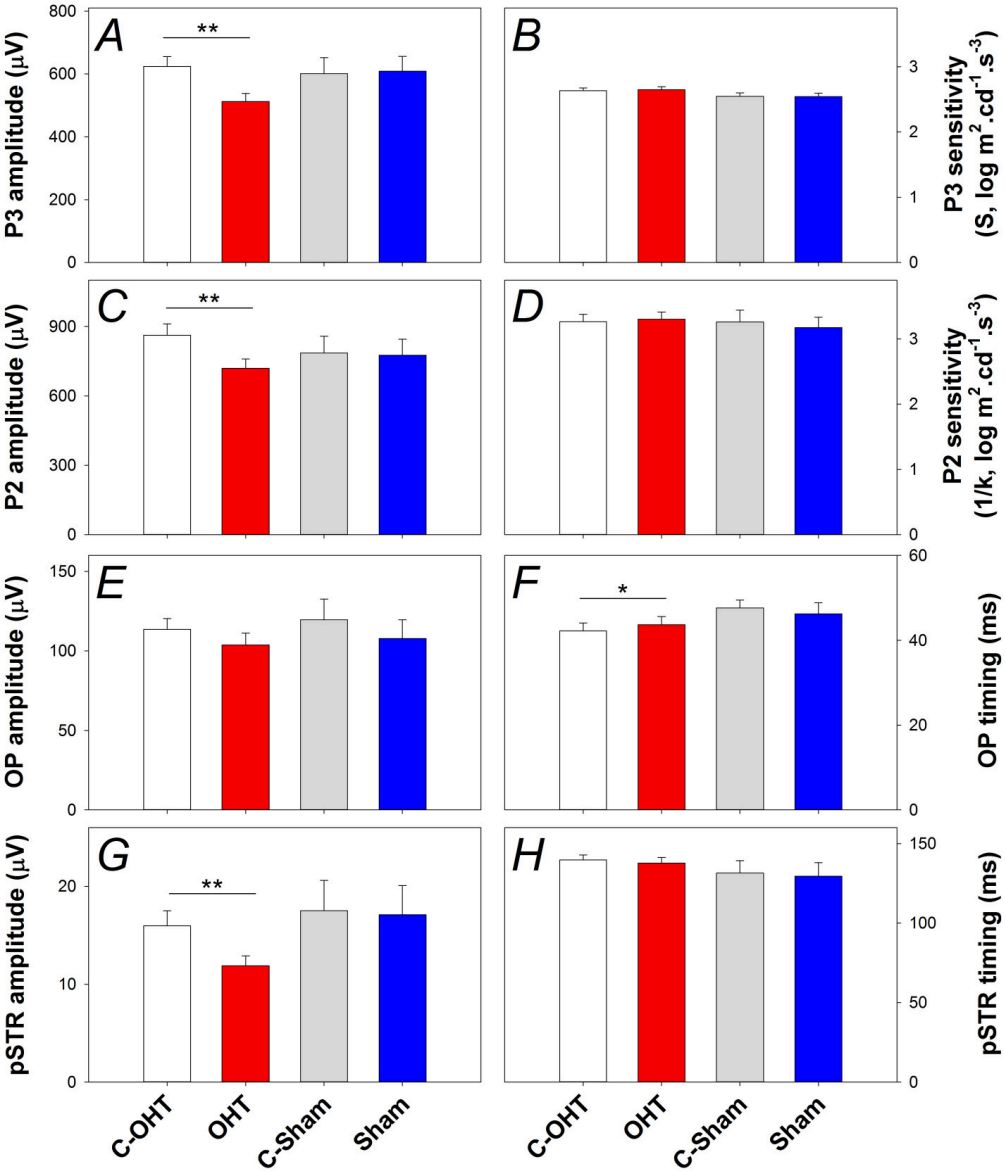
**Effect of chronic IOP elevation on retinal function. (A)** Average (± SEM) photoreceptoral (P3) amplitude for OHT (red bar, *n* = 23) and Sham (blue, *n* = 17) as well as their respective contralateral control eyes (C-OHT, unfilled; C-Sham, gray). **(B)** Photoreceptor P3 sensitivity (S). **(C)** ON-bipolar cell amplitude (P2). **(D)** ON-bipolar cell (P2) sensitivity (1/k). **(E)** Inner retinal inhibitory circuits [Oscillatory Potentials (OPs)]. **(F)** OP peak implicit time. **(G)** Ganglion cell mediated positive scotopic threshold response (pSTR). **(H)** pSTR implicit time. ^**^p < 0.001, ^*^p < 0.05.

The P3 amplitude was significantly reduced in OHT eyes (−512 ± 26 μV vs. C-OHT −624 ± 31 μV, −15 ± 4%, *p* < 0.01; Figure 5A), whereas photoreceptor sensitivity was not significantly affected (OHT 2.65 ± 0.05 log m^2^cd^−1^s^−3^ vs. C-OHT 2.63 ± 0.04 log m^2^cd^−1^s^−3^, *p* = 0.81, Figure 5B). ON-bipolar cell P2 amplitude was also significantly reduced after 12 weeks of chronic IOP elevation (OHT 719 ± 41 μV vs. C-OHT 863 ± 49 μV, 15 ± 4%, *p* < 0.05; Figure 5C), whereas P2 sensitivity was not significantly affected (OHT 3.30 ± 0.11 log m^2^cd^−1^s^−3^ vs. C-OHT 3.26 ± 0.12 log m^2^cd^−1^s^−3^, *p* = 0.82, Figure 5D). OP amplitude (OHT, 104 ± 7 μV vs. C-OHT 114 ± 7 μV, *p* = 0.1; Figure 5E) was unaffected by 12 weeks of chronic IOP elevation, nd implicit time was significantly delayed (OHT, 43.7 ± 1.9 ms vs. C-OHT 42.3 ± 1.6 ms, *p* = 0.03; Figure 5F). The largest effect of chronic IOP elevation on ERG responses was found in the RGC pSTR, which was reduced by 19 ± 6% in OHT eyes compared to control eyes (OHT, 11.9 ± 1 μV vs. C-OHT 16 ± 1.6 μV, *p* < 0.01, Figure 5G). There was no difference in pSTR implicit time (Figure 5H). The sham group showed no statistical difference between sham eyes and control eyes for any of the measured parameters (all *p* > 0.05) and control eyes in the OHT group (C-OHT) were similar to the C-Sham eyes in all parameters (all *p* > 0.05), with the exception that OP implicit time was slower (*p* = 0.05).

### The effect of chronic IOP on retinal structure

**Figure 6:**
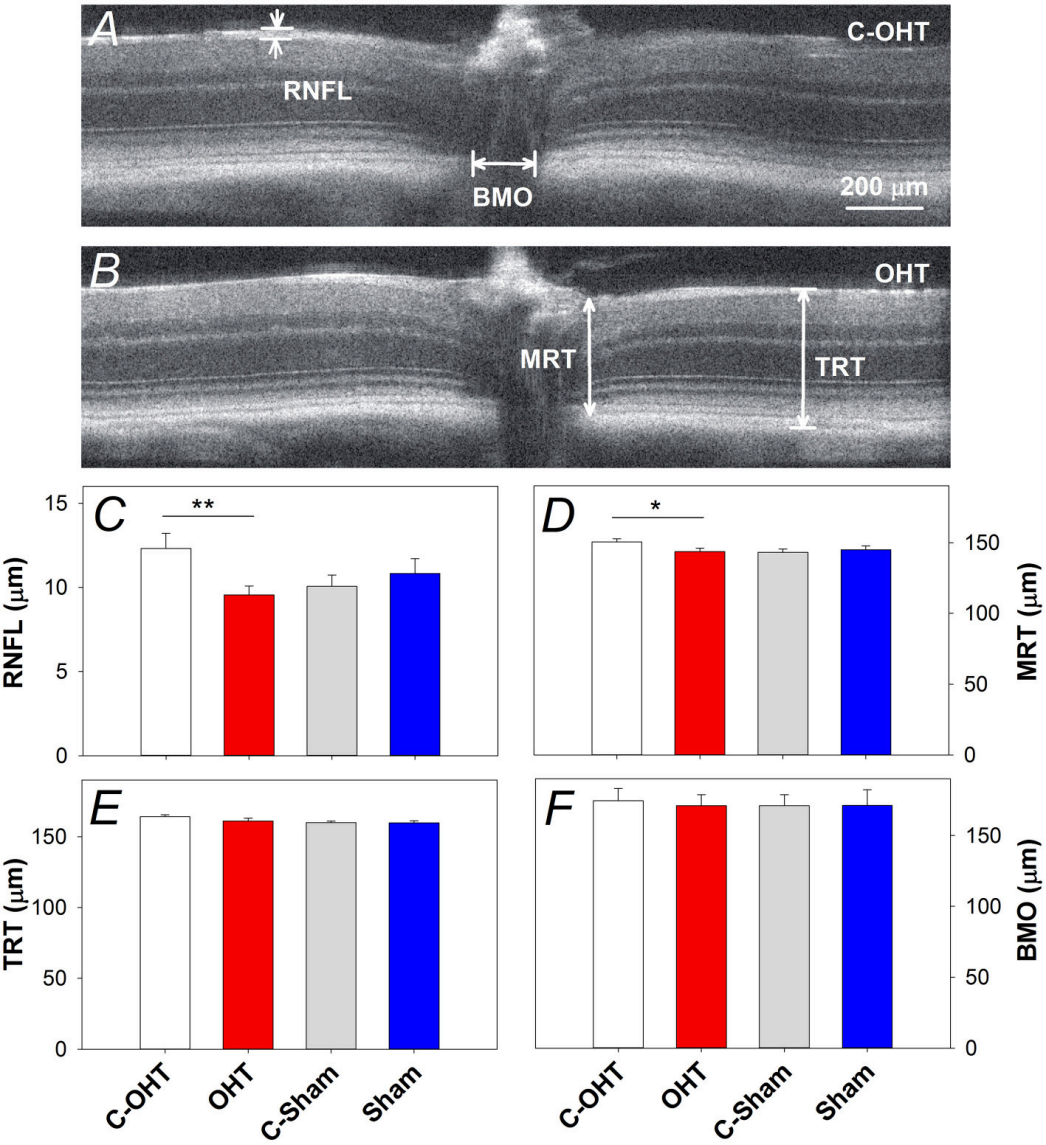
**Effect of chronic IOP elevation on retinal structure. (A,B)** Representative OCT images of a sutured eye and the control eye of a mouse in the OHT group. Bruch's membrane opening width (BMO), minimum rim thickness (MRT), total retinal thickness (TRT), and retinal nerve fibre layer (RNFL) thickness were measured on both temporal and nasal sides of the nerve and averaged. **(C)** Average (± SEM) RNFL thickness for OHT (red, *n* = 23) and Sham (blue, *n* = 17) as well as their respective contralateral control eyes (C-OHT, unfilled; C-Sham, gray). **(D)** Group average MRT. **(E)** Group average TRT. **(F)** Group average BMO. ^**^p < 0.001, ^*^p < 0.05.

Retinal structure characterized using SD-OCT is shown in Figure 6A (control eye) and Figure 6B (OHT eye) highlighting key parameters for our study. Group comparisons shows that the RNFL thickness (9.6 ± 0.5 μm vs. 12.3 ± 0.9 μm, *p* < 0.0.1; Figure 6C) and minimal rim thickness (MRT; 143.5 ± 2.5 μm vs. C-OHT 150.4 ± 2.3 μm, *p* = 0.04, Figure 6D) were significantly reduced in OHT eyes compared with control eyes. Neither total retinal thickness (TRT, Figure 6E), nor Bruch's membrane opening (BMO, Figure 6F) was significantly affected. There was no difference in any of the OCT parameters between treated and control eyes in the sham group (all *p* > 0.05), whilst comparisons of the untreated control eyes in the two groups (C-OHT vs. C-Sham) showed differences in TRT (*P* = 0.04) and MRT (*P* = 0.03).

**Figure 7:**
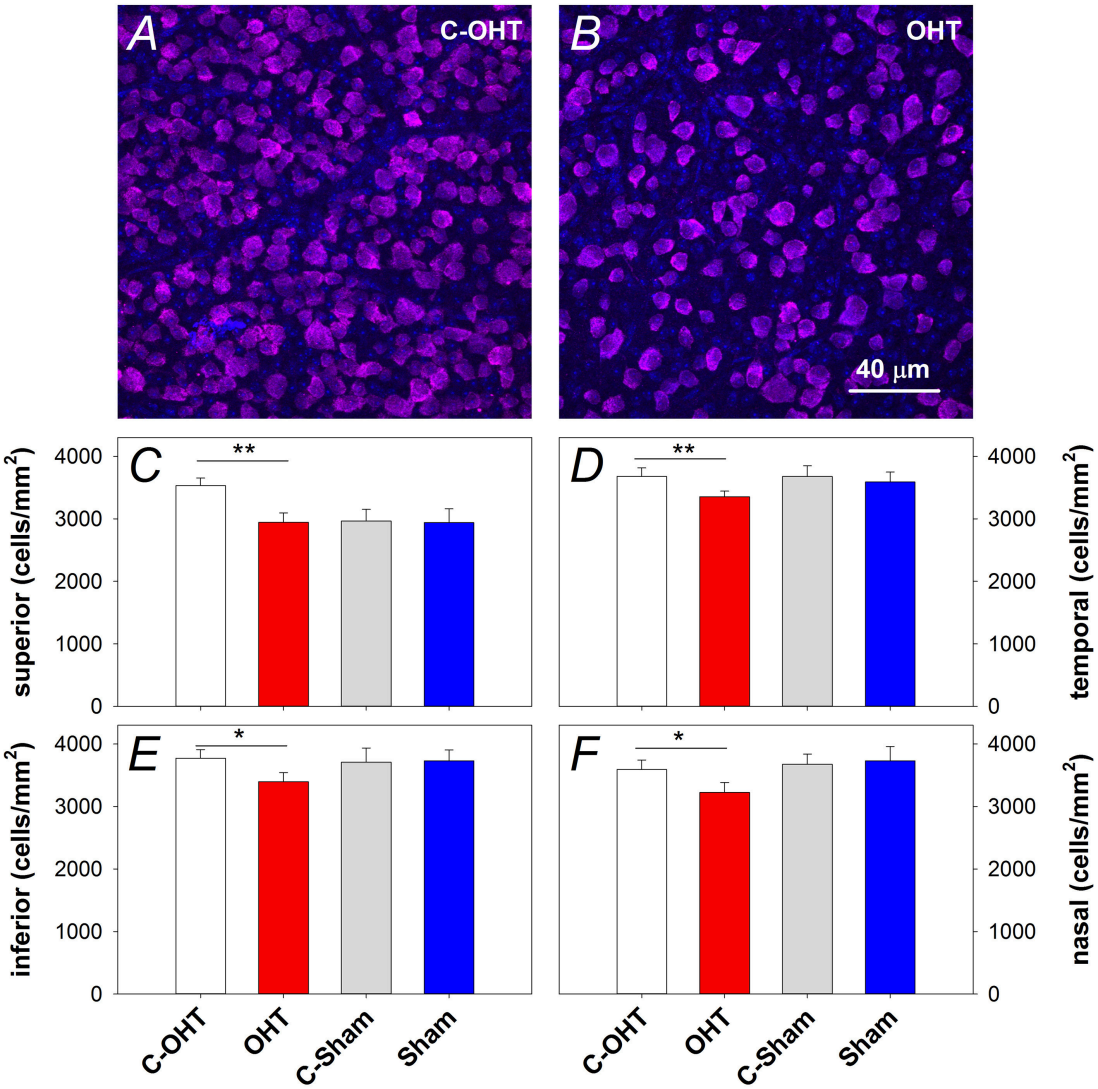
**Effect of 12 weeks of chronic IOP elevation on RGC density. (A,B)** Representative micrographs of RGCs stained with RBPMS in an OHT eye and its contralateral control eye. **(C)** Average (± SEM) RGC density on the superior retina, for OHT (red, *n* = 12) and Sham (blue, *n* = 8) as well as their respective contralateral control eyes (C-OHT, unfilled; C-Sham, gray). **(D)** RGC density in the temporal retina. **(E)** RGC density in the inferior retina. **(F)** RGC density in the nasal retina. ^**^P < 0.001, ^*^P < 0.05.

In addition to the *in vivo* structural assessments, retinal immunohistochemistry and cell counts were used to estimate the effect of chronic IOP elevation on RGC density. RBPMS immunolabeling was assessed in contralateral control and OHT eyes (Figures 7A,B respectively) after 12-weeks of IOP elevation, and showed a reduction in RGC density in all quadrants (superior: 2944 ± 123 vs. 3531 ± 122 cells/mm^2^, *p* < 0.01, temporal: 3354 ± 90 vs. 3678 ± 137 cells/mm^2^, *p* < 0.01, inferior: 3397 ± 143 vs. 3772 ± 137 cells/mm^2^, *p* = 0.02, nasal: 3221 ± 163 vs. 3592 ± 150 cells/mm^2^, *p* < 0.05). In contrast, there was no difference in RGC density in sham operated eyes compared with their controls (all *p* > 0.05; Figures 7C–F). When comparing contralateral control eyes across treatment groups, there was no alteration in RGC density in the temporal, inferior and nasal quadrants (all *p* > 0.05), however C-OHT eyes had higher RGC density in the superior quadrant compared to C-sham eyes (difference of 565 ± 215 cells/mm^2^, *p* = 0.02).

### The effect of chronic IOP elevation on stretch sensitive membrane channels within the retina

**Figure 8:**
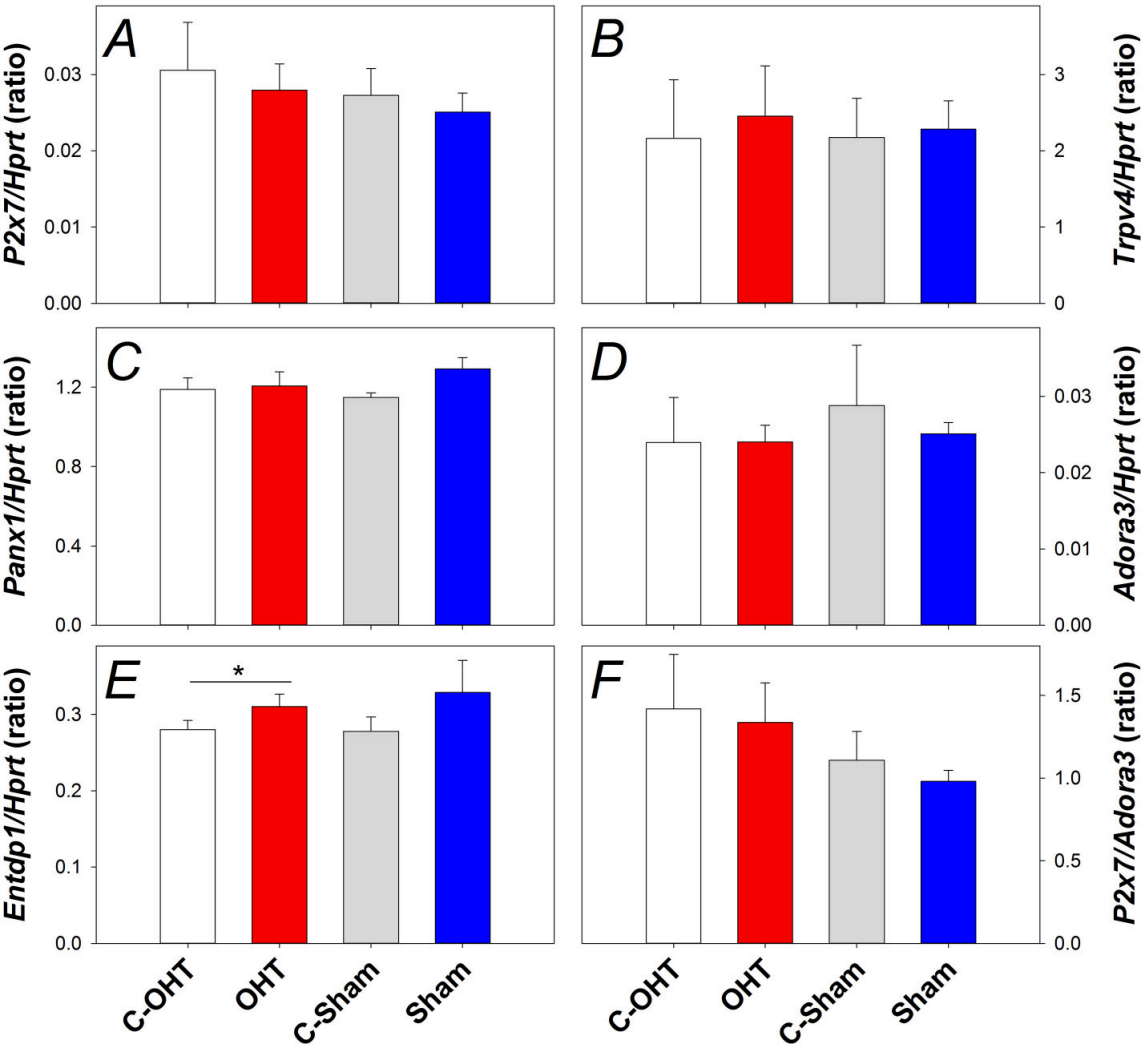
**Effect of 12 weeks of chronic IOP elevation on expression of pressure sensitive cell membrane receptors assayed 12 weeks after IOP elevation. (A)** Average (± SEM) *P2x7* gene expression relative to *Hprt* for OHT (red, *n* = 9) and Sham (blue, *n* = 9) as well as their respective contralateral control eyes (C-OHT, unfilled *n* = 7; C-Sham, gray, = 8). **(B)** Average *Trpv4* expression relative to *Hprt* (OHT *n* = 9, C-OHT *n* = 8, Sham *n* = 9, C-Sham *n* = 8). **(C)** Average *Panx1* expression relative to *Hprt* (OHT *n* = 9, C-OHT *n* = 8, Sham *n* = 9, C-Sham *n* = 8). **(D)** Average *Adora3* expression relative to *Hprt*. **(E)** Average *Entpd1* expression relative to *Hprt*. **(F):** ratio of *P2x7/Adora3*. ^*^ *p* = 0.02.

To understand the underlying mechanisms of IOP elevation induced retinal injury, gene expression of several pressure or cell membrane stretch sensitive channels was investigated, including components of the purinergic system (P2X7 and A3 receptors, EctoATPase-1), pannexin 1 *(Panx1)* and transient receptor potential cation channel subfamily V member 4 (Trpv4) channels. Figure 8 shows gene expression for *P2x7, Trpv4, Panx1, Adora3*, and *Entpd1* (Panels A–E, respectively). There was no significant difference in the gene expression of *P2x7* (Figure 8A), *Trpv4* (Figure 8B), *Panx1* (Figure 8C), or *Adora3* (Figure 8D) between OHT and their control eyes (*p* > 0.05), while *Entpd1* gene expression was significantly increased in OHT eyes (Figure 8E, 0.31 ± 0.01 vs. 0.28 ± 0.01, *p* = 0.02). The balance between the receptors, P2X7 and A3, has been suggested to be an indicator altered purinergic signaling, however, when the ratio of gene expression was investigated, no effect was evident (Figure 8F, *p* = 0.45). For all genes assessed, there was no difference between treated and control eyes in the sham group, nor between C-OHT and C-Sham eyes (all *p* > 0.05).

## Discussion

This paper describes the application of the circumlimbal suture model in mouse eyes. Immediately after suture application IOP spiked to average of 38 ± 2 mmHg (range 26–62 mmHg). This response was also observed following this procedure in rats (Liu et al., 2015, 2016). After this spike, IOP was maintained above baseline (Δ IOP = 6 ± 1 mmHg) for the next 12 weeks. Chronic elevation in IOP did not result in the development of a generalized ocular pathology, with no observable alterations seen in the anterior chamber, retinal fundus, retinal blood flow, nor total retinal thickness. Chronic IOP elevation did, however, lead to altered outer and inner retinal function, reduced ganglion cell function, RNFL thinning and an 8% reduction in RGC density.

In this study, 12 weeks of chronic IOP elevation produced a 19 ± 6% reduction in ganglion cell function (pSTR), a 17 ± 5% RNFL thinning and an 8% reduction in ganglion cell density. This level of RGC dysfunction and cell loss is comparable to that reported for other murine models of chronic IOP elevation. Frankfort et al. (2013) using intracameral injections of microbeads in mice reported that exposure to an IOP of 20 mmHg (5 mmHg above baseline) for 12 weeks resulted in a 7.4 and 11.2% reduction in RGC density at 6 and 12 week, respectively. These authors also showed that this reduction in RGC density was associated with a relative attenuation of ganglion cell function as measured using the pSTR. Other studies have also reported similar effects to our current observation. Cone et al. (2012) using injected microbeads along with a viscoelastic agent (Helon®) found a positive correlation between the IOP elevation and axonal loss over 28 weeks (~3 mmHg above baseline: 15% reduction, ~6.5 mmHg above baseline: 36% reduction). Furthermore, Sappington et al. (2010) report that IOP elevation to 20 mmHg (5 mmHg above baseline) for 4–5 weeks resulted in a 20% reduction of axons. Despite following our model out for 12 weeks, the level of functional deficit was much less than the 20% outer retinal and 50% ganglion cell deficit reported after 7 weeks in the episcleral vein laser ablation model (Holcombe et al., 2008). This significant pathology reflects the possibility that the episcleral vein ablation approach induces a significant inflammatory response in addition to IOP elevation.

Similar to the microbead approach (Sappington et al., 2010), the circumlimbal suture model recapitulates some characteristics seen in human glaucoma. This model produced clear RGC loss, as evidenced by histological cell counts and loss of RNFL on OCT measurements. Furthermore, the OCT data suggests the cell loss to be specific for ganglion cells since total retinal thickness was not significantly affected. Our Doppler blood flow imaging data supports the suggestion that this ganglion cell injury arises from the effects of IOP and not due to direct damage to the inner retinal blood supply. Although it is possible that the circumlimbal suture compromises choroidal blood flow, the lack of a more severe outer retinal deficit (ERG and OCT) suggests that this does not occur to any significant extent. The mechanism by which IOP remains elevated across the 12 weeks in this model does not involve posterior or anterior synechia, nor angle closure. A likely mechanism by which IOP is elevated is via increased episcleral venous pressure, and thus reduced aqueous outflow, which makes it more akin to the episcleral venous scarring model (Moore et al., 1995; Ruiz-Ederra and Verkman, 2006) or laser ablation of the episcleral veins (Holcombe et al., 2008). However, unlike these models, the circumlimbal suture model has little inflammation (Liu et al., 2015).

We show that 12 weeks of chronic IOP elevation leads to a significant increase in gene expression for *Entpd1*. This change is in agreement with the recent work of Lu et al. (2015) who show that *Entpd1* upregulation is a consistent finding following chronic IOP elevation in three laboratory species. In particular, the authors employ the Tg-MYOC^Y437H^ mouse (assessed at 40 weeks, gradual increase of 4 mmHg IOP elevation from week 20) model, the rat episcleral scarring model (assessed at 15 days, ~10 mmHg IOP elevation) and the non-human primate laser model assessed at 18 weeks, 15 mmHg IOP elevation. They find that this elevated expression of *Entpd1* is associated with an increase in extracellular ATP. NTDPase1 (the Entpd1 protein product) is an ectoenzyme that dephosphorylates ATP and thus upregulation of the *Entpd1* gene is likely to reflect an adaptive response to cope with an increase in extracellular ATP associated with chronic IOP elevation (Perez de Lara et al., 2015). Breaking down eATP to ADP reduces the potential for detrimental activation of P2X7 receptors (Zhang et al., 2005; Resta et al., 2007), as well as enhancing protective effects mediated through adenosine receptors (A1 and A3) (Zhang et al., 2006).

Unlike the IOP-induced increase in *Entpd1* expression, no alteration in *P2x7* or *Adora3* (A3 receptor) gene expression was observed in this study. To our knowledge the expression of purinergic receptors (*P2x7, Adora3*) have not been assessed following chronic IOP elevation. Despite this, there is growing evidence that changes in purinergic signaling may be an important mechanism of RGC death in glaucoma. Elevated levels of extracellular ATP has been documented in clinical samples from angle closure glaucoma patients (Zhang et al., 2007), as well as in models of chronic IOP elevation (Lu et al., 2015). Furthermore, in isolated tissue preparations (Reigada et al., 2008) excessive ATP via P2X7 triggers calcium mediated RGC death (Zhang et al., 2005; Resta et al., 2007). Following acute IOP elevation (1 h, 90 mmHg), *P2x7* receptor expression showed transient changes, increasing 1 day after IOP insult, yet returning to baseline levels by day 3 (Sugiyama et al., 2013). Similarly, following optic nerve crush, *P2x7* upregulation peaked at day 3 returning to baseline after 7 days (Kakurai et al., 2013). It is possible that a transient change in purinergic receptor gene expression may have been missed in the current study, where expression levels were assessed only after 12 weeks of IOP elevation. Additionally, unlike the aforementioned studies, the insult induced in this study was mild (~5 mmHg above baseline) and thus may not have been severe enough to induce a change in purinergic receptor expression.

We also did not find a significant change in expression of any of the genes associated with stretch sensitive channels (Trpv4, Panx1). There have been few studies examining the effects of chronic IOP elevation. One study employing the Tg-MYOC^Y437H^ mouse model of chronic glaucoma has reported an upregulation of pannexin 1, 2, and 3 in 8 months old animals (Beckel et al., 2014). One possible reason for the discrepancy could be the age of animals used. The age of mice assessed by Beckel et al. was several months older than the mice used in our study, and at that stage there appears to be more severe ganglion cell loss in the Tg-MYOC^Y437H^ model (Zode et al., 2011). Furthermore, age-related increases in *Panx1* (Mawhinney et al., 2011) and *Trpv4* (Lee and Choe, 2014) expression have been documented in the central nervous system of rats. Thus, age-related differences may be important in determining the time course and extent of changes in stress-related channels in response to chronic IOP elevation. Furthermore, stress channels often exhibit transient responses in expression levels. Ganglion cells exposed to hydrostatic pressure shows changes in *Trpv* channel expression at 24 h, but not at 48 h (Sappington et al., 2015). Similarly, Weitlauf et al. (2014) show that following the onset of chronic IOP elevation, changes to TRPV1 are elevated at 1 and 3 weeks but return to normal at 5 and 7 weeks. Thus, it may be that ATP and adenosine receptors as well as stretch sensitive channel expression had normalized by week 12 in our model. It should be noted that our analysis of this pathway is limited to the expression of a small number of genes at a single time point. The significance of and time course of changes in transcript levels need to be confirmed using complementary approaches, including assessment of protein levels for example by immunohistochemistry and/or western blot techniques.

The presence of the IOP spike immediately after model induction is noteworthy, as such spikes do not occur in human glaucoma. However, similar IOP spikes have been observed in various inducible models of chronic IOP elevation in mice (Holcombe et al., 2008; Cone et al., 2012). The level of IOP reached during the spike may impact the susceptibility of the retina to the subsequent chronic IOP elevation. However, in our sham group, which experienced similar acute IOP spike as the OHT group but were not chronically exposed to IOP (suture removed day 2), were similar to the untreated contralateral controls and did not exhibit any significant changes to structure, function or histology. Thus, the changes seen in the OHT group likely reflect the chronic IOP components of injury, rather than the acute IOP elevation. This would also indicate that the IOP spike associated with model induction was of a magnitude and duration that is within the capacity for the retina and RGCs to fully recover from. Complete functional recovery of the ERG from acute IOP spikes that were of a similar magnitude have been reported in both rats (He et al., 2008, 2006; Bui et al., 2013; Lim et al., 2014) and mice (Kong et al., 2009). In rats we have recently shown that suture removal and IOP lowering afford complete functional recovery (Liu et al., 2015, 2016). Suture removal was used in this study as a control; this means that this simple intervention can be used to lower IOP at any stage during the neurodegenerative process. This would allow the process of recovery or neuroplasticity to be studied in transgenic mouse models in a way not previously possible with other inducible models.

## Conclusions

We show that IOP can be chronically elevated in mouse eyes using a circumlimbal suture. We characterize this novel model to show that ocular hypertension results in mild retinal dysfunction, RNFL thinning and a reduction in RGC density along with an increased *Entpd1* gene expression. This inducible model produces retinal changes similar to that observed in primary open-angled glaucoma, without many of the drawbacks inherent in other murine models of chronic IOP elevation.

## Author contributions

DZ, CN, ZH, AV, and BB: Study design, data collection, data analysis, manuscript preparation. VW, JL, AJ, and HC: Data collection, data analysis, manuscript preparation. EF: data analysis, manuscript preparation.

## Funding

National Health and Medical Research Council of Australia project grant (1046203), Australian Research Council Future Fellowship (FT130100338).

### Conflict of interest statement

The authors declare that the research was conducted in the absence of any commercial or financial relationships that could be construed as a potential conflict of interest.
